# Detailed spatial characterization of superficial hip muscle activation during walking: A multi-electrode surface EMG investigation of the gluteal region in healthy older adults

**DOI:** 10.1371/journal.pone.0178957

**Published:** 2017-06-05

**Authors:** Christoph Anders, Steffen Patenge, Klaus Sander, Frank Layher, Uta Biedermann, Raimund W. Kinne

**Affiliations:** 1 Division of Motor Research, Pathophysiology and Biomechanics, Clinic for Trauma, Hand and Reconstructive Surgery, Jena University Hospital, Jena, Germany; 2 Chair of Orthopedics, Department of Orthopedics, Jena University Hospital, Waldkrankenhaus "Rudolf Elle", Eisenberg, Germany; 3 Institute of Anatomy I, Jena University Hospital, Jena, Germany; 4 Experimental Rheumatology Unit, Department of Orthopedics, Jena University Hospital, Waldkrankenhaus "Rudolf Elle", Eisenberg, Germany; Duke University, UNITED STATES

## Abstract

**Purpose:**

A multi-electrode array was used to generate spatially resolved Surface electromyography (SEMG) data of the hip muscles in healthy older adults. The cohort was meant to serve as an age-matched, normal control population for future surgical and rehabilitative studies in patients undergoing total hip arthroplasty, in view of the large, continuously increasing number of hip joint replacements.

**Methods:**

Bilateral hip muscle SEMG activity, including tensor fasciae latae (TFL), gluteus medius (Gmed), and gluteus maximus (Gmax), was measured during locomotion on a walkway at self-selected slow, normal, and fast walking speeds (age-matched cohort of 29 females and 25 males). Eight equally-spaced, vertically oriented bipolar channels were applied on a horizontal line at mid-distance between iliac crest and greater trochanter (length 17.5 cm; named P1 to P8). Time-independent parameters (e.g., mean amplitude) were derived from the amplitude curves expressed as root mean square.

**Results:**

The acquired SEMG data were not significantly influenced by gender (p = 0.202) or side (p = 0.313) and were therefore pooled. The most ventral to central electrode positions P1 to P5, representing TFL and ventral to central Gmed, showed the highest mean amplitude levels (averaged over the whole stride; 0.001 < p < 0.027 against P6 to P8; Bonferroni-adjusted paired t-test) at all walking speeds. Also, the respective curves showed two distinct amplitude peaks (representing load acceptance and hip stabilization during mid-stance), with a continuous increase of the first peak from P1 to P4 (most pronounced at fast speed) and the second peak from P1 to P3. Independently of the underlying individual muscles, both peaks displayed a continuous time shift from the most dorsal P8 to the most ventral P1 position, with the peaks for the ventral positions occurring at later time points during the normalized stride.

**Conclusions:**

The continuously changing activation patterns of the superficial muscles in the gluteal region during walking may reflect function-driven, finely tuned coordination patterns of neighboring muscles and muscle segments, rather than independent activation of anatomically defined muscles. This may be important for the definition of specific target parameters for the improvement and/or normalization of muscle function during training and post-injury rehabilitation.

## Introduction

The human hip joint, a large, heavily loaded joint, requires strong muscular support for adequate functioning. During walking, muscular prevention of subsidence of the contralateral hip throughout the stance phase is of utmost importance [1]. This function is chiefly executed by the gluteus medius (Gmed), but also involves a combined effort of the gluteus maximus (Gmax), gluteus minimus, and tensor fasciae latae muscles (TFL) [1–5] and is clearly modified by walking speed [3, 6, 7]. These muscles build a powerful triangular ensemble spanning between the anterior superior iliac spine (ASIS), the posterior superior iliac spine, and the greater trochanter region of the femur. Due to the large dimension covered by these muscles, spatially differentiated and changing functional characteristics are to be expected during locomotion, likely resulting in a spatially heterogeneous Surface EMG (SEMG) signal both among and within individual muscles. Indeed, the dorsal Gmed segments and the Gmax play a more prominent role for powerful propulsion upon extension of the leg during the stance phase of the gait cycle (and its external rotation), whereas the ventral Gmed parts, together with the TFL, are more important for the flexion (and internal rotation) during the swing phase of the gait cycle [1]. Also, anatomic spatial heterogeneity has been reported for the Gmed, for which three [8, 9] or even four [8] separately innervated segments were identified.

Despite this heterogeneity across the Gmed, current internationally accepted guidelines [10] only recommend one centrally located electrode pair for SEMG investigations of each of the superficial hip muscles TFL, Gmed, and Gmax (www.seniam.org). In the only published report on spatially differentiated activation of individual Gmed segments during locomotion [11], intramuscular needle EMG was used, which, due to its invasive character, may be less broadly applicable in orthopedics, physiotherapy, and rehabilitation studies [12]. In their study, Semciw et al. [11] found evidence for segmental activation of the anterior Gmed during the stance phase of the gait cycle, as reflected by the fact that the first and second activity peak of the anterior Gmed occur significantly later than the respective peaks of the middle and (posterior) segments. Thus, a spatially resolved SEMG signal of the gluteal region may reflect the activation of both anatomically defined individual muscles (i.e., TFL, Gmed, and Gmax; [3]) and/or separately innervated muscle subregions [8, 9]. The time delay of the different activity peaks within the Gmed led us to design a study based on an extended electrode arrangement to also capture the fine coordination patterns among individual muscle segments.

The cohort investigated in the present study was intended as an age-matched, normal control population for future surgical and rehabilitative studies in older patients with total hip arthroplasty (THA), the most frequent joint replacement surgery method [13, 14]. The frequently used anterolateral minimally-invasive surgery (MIS) approach for THA inevitably leads to traumatization of the anterior parts of the Gmed, whereas the posterior MIS approach, though technically more challenging, avoids damage to the Gmed [15] and only requires splitting of the Gmax. Gmed-sparing THA approaches are therefore particularly relevant for full restoration of a normal and pain-free gait with physiological hip muscle function during rehabilitation [14–18]. Assessment of conservative and surgical interventions to treat hip dysfunction would profit from the establishment of spatially resolved measurement protocols (using several electrodes across the gluteal region) and the existence of normal control data. Protocols resulting in such normative data may help in maintaining or restoring pivotal hip function in older adults and thus counteract the severe complications associated with loss of ambulation [19–21].

SEMG or needle EMG analyses of motor control have shown that muscles and/or muscle segments act together in a functionally driven manner to meet the demands of imposed motor tasks [22, 23]. The continuing multidisciplinary effort to understand motor control has built a picture of a finely tuned network of highly synchronized patterns, at times dependent and at times independent of central nervous system control, and feedback loops [22]. In one of the most comprehensive studies of muscles involved in walking, in fact, Ivanenko et al. [24] identified five basic activation patterns between leg, hip, and torso muscles that were used repeatedly throughout gait. This study was helpful in establishing the existence of interaction patterns between different individual muscles during ambulation. The next step in understanding coordination during locomotion is to include electrode positions that represent both entire muscles and their individual functional segments. However, there is a general scarcity of studies which combined between and within muscle EMG measurements during particular motor tasks.

### Purpose, hypothesis, and design

The study was specifically designed to determine whether an expanded electrode arrangement of SEMG electrodes could allow for non-invasive, highly detailed, and spatially resolved characterization of the muscle activations across the gluteal region and whether this systematic approach would be useful in assessing its potential relevance for future surgical and rehabilitative studies in older patients.

The hypothesis of the present study was that the performance of the complex motor task of walking requires highly coordinated activation patterns of neighboring hip muscles and muscle segments rather than independent activation of anatomically defined muscles. Therefore, we expected gradually changing, but not fundamentally differing activation patterns across the gluteal region representing the finely tuned, changing functional demands on all local muscles and/or their subregions.

Electromyographical gluteal activity was quantified using on one hand the commonly applied root mean square (rms) of the amplitude, but also the cumulative muscle activity per distance (CMAPD [25, 26]). These parameters were chosen since they reflect both the absolute activity level and the activity in direct relationship to the traveled distance.

The novel aspects of the present study are thus: i) the high spatial resolution of the activity of gluteal region; and ii) the detailed statistical (and not only descriptive) analysis of the amplitude curves for every single time point of the normalized stride, allowing in depth consideration of the effects of position and walking speed.

## Methods

Fifty-four healthy age-matched subjects of both genders were enrolled in the study. Written informed consent was obtained from all subjects following detailed information about the study purpose and procedure. The study was approved by the ethics committee of the Jena University Hospital (3002-12/10). All subjects were clinically examined and briefly interviewed about their medical history. Normal mobility of the hip joint was verified by application of the neutral zero method (i.e. joint mobility from the normal anatomical 0-position towards the maximal positions concerning extension/flexion, abduction/adduction, as well as external and internal rotation [27]). Exclusion criteria were relevant orthopedic and neurologic disorders, endoprosthetic joint replacement of either the knee or the hip joints, pain during locomotion, and/or clinical signs of knee or hip osteoarthritis.

### Gait analysis procedure

After instrumentation (see below), subjects were asked to complete ten to fifteen trials on a 10 m walkway at their self-selected slow (instruction: "walk intentionally slow"), normal ("walk at your preferred walking speed"), and fast ("walk intentionally fast, like catching a bus") walking speeds. For each speed, a minimum of 50 strides at steady-state conditions (i.e., after the initial acceleration, but before the final deceleration) were used for the subsequent SEMG analysis. From these trials, selected gait data were determined (i.e., cadence, stride length, and walking speed [28]). The subjects were informed about the investigation procedure and were asked to wear their own comfortable shoes.

### SEMG measurement and processing

**Fig 1:**
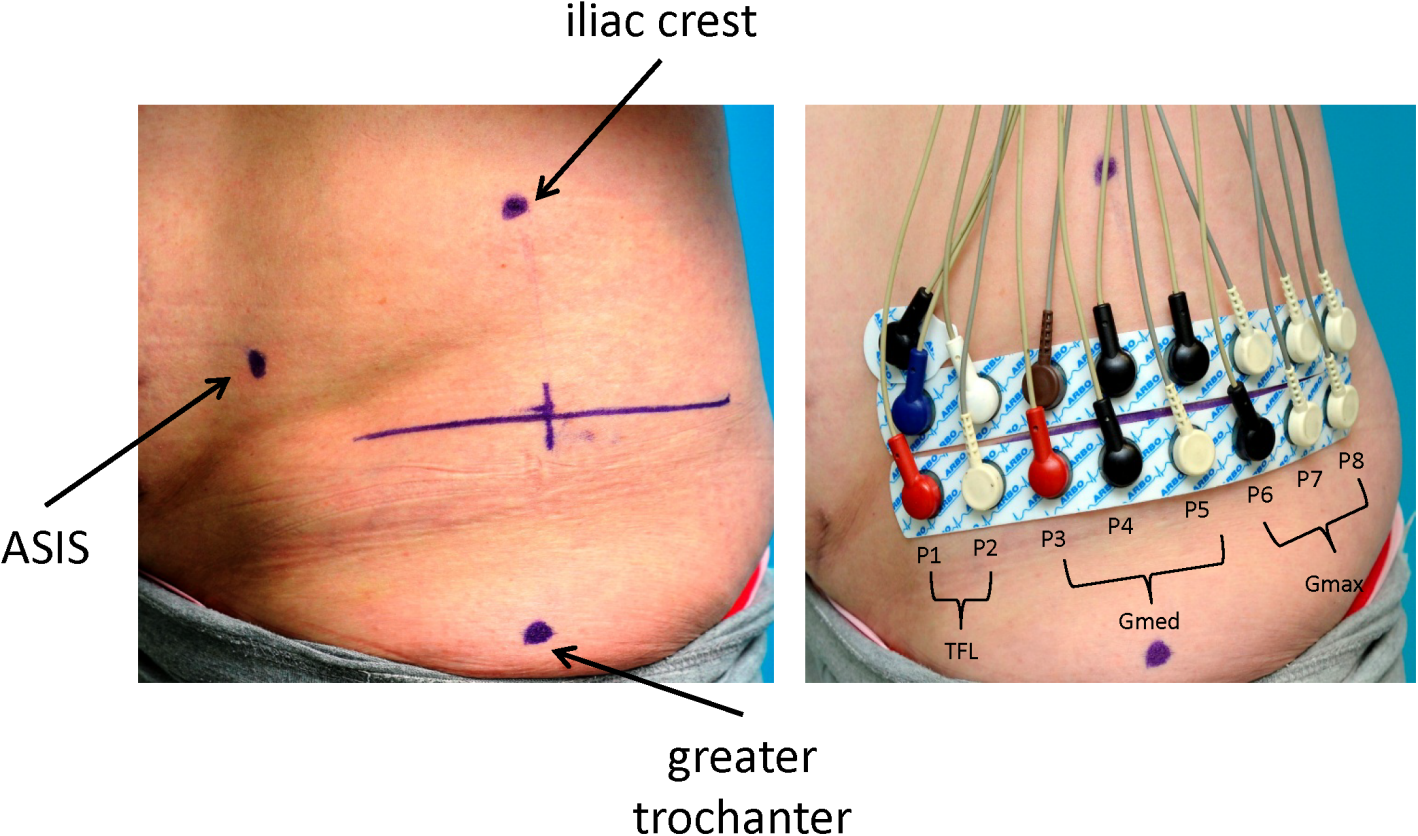
Application of the SEMG electrodes. Left panel: Labelling of the respective landmarks (left view) greater trochanter, iliac crest, anterior superior iliac spine (ASIS). Right panel: Applied electrode strips with the respective cables. Electrode positions are labeled P1 to P8 and related to the respective gluteal muscles TFL, Gmed, and Gmax. The separate electrode is one of the two conjoined bilateral electrodes above the respective ASIS serving as reference electrodes.

Analog SEMG signals were captured by applying a monopolar montage with 16 electrodes per body side. Disposable Ag-AgCl solid gel electrode strips were used (H938SG, Covidien, Germany; circular uptake area of 2 cm^2^; eight electrodes each; inter-electrode distance (IED) of 2.5 cm). In line with the SENIAM recommendations for the Gmed, a horizontal marker line was placed at mid-distance of and at a 90° angle to the vertical line between the greater trochanter and the iliac crest. The two electrode strips were then horizontally aligned immediately above and below this marker line (vertical inter-electrode distance of 2.5 cm), with their center on the vertical line between these two anatomical landmarks (Fig 1).

Conjoined bilateral electrodes at the anterior superior iliac spine (ASIS) served as reference electrodes. In order to unequivocally identify heel contacts, pressure sensors were placed below both heels.

SEMG signals were amplified with a gain of 1000 (-3 dB at 5 Hz and 700 Hz, DeMeTec, Germany), analog-to-digital converted (Tower of Measurement—ToM; 2048 samples/s, amplitude resolution: 24 bit at ± 5 V, anti-aliasing filter at 1024 Hz, DeMeTec, Germany), and stored on hard disk for subsequent offline analysis using the ATISArec capturing program (GJB, Germany).

The monopolar signals were first converted to bipolar signals by separately subtracting the signals of the corresponding electrodes of the two strips on each body side. This resulted in a bilateral arrangement of eight vertically oriented bipolar channels (named P1 to P8 from the ventral to the dorsal margin) covering a horizontal distance of 17.5 cm. For this electrode arrangement, positions P1 and P2 represented TFL activity [29], positions P3 to P5 the Gmed [30], and the three dorsal positions P6 to P8 the Gmax [31, 32] with high certainty, whereas the electrode positions P1, P4/P5 (mean value), and P8 most closely represented the respective SENIAM positions. The anatomical relationship between these electrode positions and the respective muscles was further confirmed by own detailed studies of human cadaver specimens (S1 Fig). The large area covered by the electrode positions P1 to P8 and their inter-electrode spacing were specifically chosen to address not only functional differences between the respective anatomically defined muscles, but also potential coordination patterns of neighboring muscles and their respective muscle segments [2–4]. The ventral to dorsal orientation of the electrode strips was chosen, since systematic amplitude differences in proximal to distal orientation were less likely due to the fusiform shape of the underlying muscles.

SEMG data were band-pass filtered between 20 Hz and 400 Hz. To account for the possibility of randomly occurring interferences from the electrical current supply, a 50 Hz (band width ± 1 Hz) notch filter was applied.

In order to exclude acceleration or deceleration effects and to reduce speed variability among trials, for each of the three self-selected walkway speeds only steady-state strides were considered for analysis. This was ensured by excluding all strides deviating in their stride time by more than 10% from the median time of all determined strides. Finally, outliers due to stumbles or technical artefacts resulting in irregular SEMG curve patterns (i.e., exceeding the limits of two standard deviations from the mean) were identified by visual inspection and deleted.

The signals of the remaining strides were quantified as root mean square (rms) values, smoothed with an overlapping, sample wise moving rectangular window of 50 ms (overlap 49.5 ms), and time normalized (100% = one complete stride) on the basis of two subsequent ipsilateral heel strikes (time resolution of 0.5%, 201 time points). This normalization serves to compensate for differences in stride times among strides in different subjects, trials, and at different speeds [33].

The data were then separately averaged for every individual, walking speed, bipolar channel, and time point. The resulting grand averaged amplitude curves were used to calculate the mean amplitude (i.e., average value of the normalized stride; time-independent parameter). To address the energy expenditure to walk a given distance, the cumulative muscle activity per distance (CMAPD) was calculated by dividing the respective individual mean amplitude by the walking speed that was determined using an instrumented walkway equipped with force plates (i.e. CMAPD = A/v where A is the mean amplitude in μV and v the walking speed in m/s [25, 26]). To analyze amplitude differences due to either bipolar channel positions and/or subject-specific characteristics, the grand averaged amplitude curves were also separately normalized to the respective maximum value for every subject, side, walking speed, and bipolar channel position. All these steps were performed applying custom-made scripts in the MATLAB environment (The Mathworks, USA).

During the stance phase, two additional parameters were analyzed: i) ratio between amplitude peak I just after heel strike (i.e., maximum value for each subject within the time window from 0% to 15% of the normalized gait cycle) and amplitude peak II during mid stance (25% to 40%); and ii) amplitude drop between the two amplitude peaks (i.e., minimum value for each subject between 12.5% to 37.5% of the stride; normalized to the first amplitude peak). The two amplitude peaks are the SEMG equivalents of the forces required to: i) compensate for the heel strike impact; and ii) stabilize the hip [33], respectively. All these parameters were separately extracted for every walking speed and position. The toe-off time was set at 62.5% of the normalized stride according to published data [33].

Since exclusively intra-individual comparisons were performed, normalization procedures as recommended for inter-individual comparisons of SEMG data (i.e., due to the large variability of SEMG amplitudes among subjects) were not applied in the present study.

### Statistics

#### Global statistics

A repeated measures ANOVA was performed (IBM SPSS Statistics, V21, IBM, USA) to assess statistical differences among the time-independent parameters mean amplitude and CMAPD concerning the influence of side (2 classes), speed (3), position (8), and gender (2). This also included effects on the amplitude drop and the ratio between the observed amplitude peaks during the stance phase. From the group of the above-mentioned main effects, only speed and position reached a level of p ≤ 0.05 and were thus further considered.

#### Statistics of time-dependent data

Paired t-tests were performed to assess statistical effects of position or speed on the parameters amplitude and normalized amplitude for every time point throughout the gait cycle. In order to correct these test results for the effects of multiple testing (in particular caused by the high number of time points), the stepwise Bonferroni-Holm procedure was applied [34] to avoid the accumulation of a type I statistical error, i.e., to falsely reject the respective null hypotheses. A detailed description of the Bonferroni-Holm procedure can be found in the supplementary material (S1 Text).

#### Multiple testing corrections for speed and position comparisons

An additional common Bonferroni correction was subsequently performed to further address the problem of multiple testing for different positions or walking speeds. In detail, eight positions had to be considered for position-related differences, resulting in yet another correction of the statistical significance level α for 28 pairwise comparisons (resulting in a least required final p level of 8.9*10^−6^). For walking speed-related differences, the three correction levels resulted in a least required final p level of 8.3*10^−5^.

## Results

### Patient cohort

As expected, the age-matched females and males in the present study significantly differed concerning their height, weight, and BMI (Table 1).

**Table 1:** Subject characteristics.

Parameter	Female (n = 29)	Male (n = 25)	t-test
Age [years]	63.7 ± 6.8	61.3 ± 7.0	n.s.
Height [cm]	164.0 ± 5.9	178.3 ± 6.5	<0.001
Weight [kg]	68.8 ± 11.5	90.2 ± 12.2	<0.001
BMI [kg/m^2^]	25.5 ± 4.4	28.3 ± 3.5	0.010

BMI: body mass index; data are displayed as means ± SD; n.s.: not significant

### Gait data

Cadence and step length showed expected and significant differences between genders (all p values ≤ 0.01) except for the cadence at slow walking speed; however, walking speeds in females and males were virtually identical (Table 2).

**Table 2:** Global gait data.

Parameter	Walking speed	Female (n = 29)	Male (n = 25)	Pooled (n = 54)
Cadence^1^	Slow	0.63 ± 0.07	0.63 ± 0.06	0.63 ± 0.07
	Normal	**0.86 ± 0.05** ^**§**^	**0.81 ± 0.04** ^**§**^	0.84 ± 0.06 ^§^
	Fast	**0.94 ± 0.06** ^**§**^ ^**$**^	**0.88 ± 0.06** ^**§**^ ^**$**^	0.91 ± 0.07 ^§^ ^$^
Step length [m]	Slow	**0.55 ± 0.05**	**0.61 ± 0.07**	0.58 ± 0.07
	Normal	**0.70 ± 0.07** ^**§**^	**0.75 ± 0.06** ^**§**^	0.72 ± 0 07 ^§^
	Fast	**0.75 ± 0.07** ^**§**^ ^**$**^	**0.84 ± 0.07** ^**§**^ ^**$**^	0.79 ± 0.08 ^§^ ^$^
Walking speed [m/s]	Slow	0.87 ± 0.15	0.91 ± 0.18	0.89 ± 0.16
	Normal	1.47 ± 0.18 ^§^	1.44 ± 0.20 ^§^	1.46 ± 0.17 ^§^
	Fast	1.73 ± 0.21 ^§^ ^$^	1.74 ± 0.21 ^§^ ^$^	1.74 ± 0.21 ^§^ ^$^

^1^ Cadence values appear as relative values referred to body height [28].

Significant differences (all p values ≤ 0.01) between genders appear as bold numbers.

§ p < 0.001 vs. slow

$ p < 0.001 vs. normal. Data are displayed as means ± SD.

All three parameters showed statistically significant differences among the three self-selected speeds slow, normal, and fast (all p values < 0.001).

### Global statistical analysis

For the time-independent parameters mean amplitude and CMAPD there was no statistically significant influence of gender or side. In contrast, there was a highly significant influence of walking speed and position on all parameters (all p values < 0.001). Therefore, the final ANOVA design included only walking speed and position as the remaining variables and considered gender-independent data, as well as averaged values for the left and right side of every individual (n = 54). This ANOVA indicated a strong influence of position and walking speed on the time-independent parameters as well as an interaction of position and speed (all p values ≤ 0.001, Table 3).

**Table 3:** ANOVA results for walking speed and position-related effects (data for gender and side are pooled).

Parameter		Position	v	Position * v
Mean amplitude	p value	<0.001	<0.001	0.001
	F value	10.675	22.73	3.360
CMAPD	p value	<0.001	<0.001	<0.001
	F value	10.526	37.220	5.770

v: walking speed; CMAPD: cumulative muscle activity per distance

Gender-independent and side-averaged data were then also used for the statistical analysis of the time-dependent variables amplitude and normalized amplitude.

### Time-independent SEMG parameters

#### Position-dependent effects

**Fig 2:**
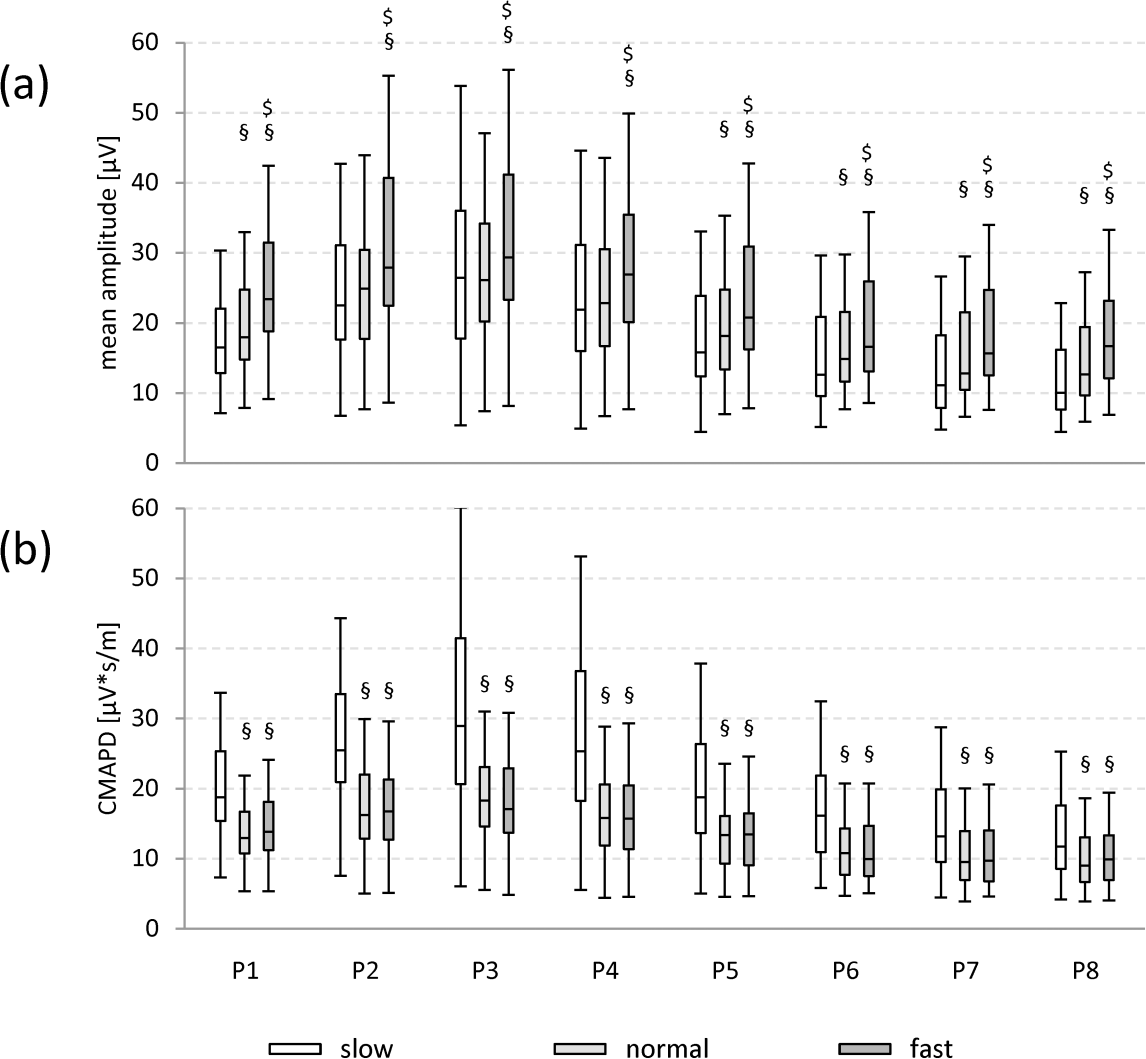
Boxplots of mean amplitude and CMAPD. Mean amplitude(a) and CMAPD (b) for all subjects regarding the different electrode positions and walking speeds. Data are pooled for gender and side. Significant differences among the different walking speeds are indicated for the individual electrode positions P1-P8: § vs. slow, $ vs. normal. All p values are < 0.05 (Bonferroni corrected).

Irrespective of the walking speed (slow, normal or fast), mean amplitude and CMAPD increased from the most ventral position P1 to position P3 (relative increase of mean amplitude at slow: 38%, normal: 31%, fast: 20%; CMAPD at slow: 35%, normal: 29%, fast: 19%) and were generally higher in the ventral positions (P1-P5) than in the dorsal ones (P6-P8; relative difference of mean amplitude at slow: between 20% and 62%, normal: 17% to 52%, fast: 20% to 47%; CMAPD at slow: 14% to 60%, normal: 16% to 51%, fast: 17% to 43%; Fig 2A and 2B; S2A and S2B Fig).

Using post hoc tests for pairwise comparisons of individual electrode positions, significant differences were observed among almost all positions concerning mean amplitude and CMAPD (Table 4).

**Table 4:** Post hoc differences for the time-independent parameters among individual electrode positions (p values including Bonferroni correction; independent of the walking speed).

Parameter		P2	P3	P4	P5	P6	P7	P8
Mean amplitude	P1	<0.001	<0.002	n.s.	n.s.	0.026	<0.001	<0.001
	P2		n.s.	n.s.	0.003	<0.001	<0.001	<0.001
	P3			<0.001	<0.001	<0.001	<0.001	<0.001
	P4				<0.001	<0.001	<0.001	<0.001
	P5					<0.001	<0.001	<0.001
	P6						0.01	n.s.
	P7							n.s.
CMAPD	P1	<0.001	0.001	n.s.	n.s.	n.s.	<0.001	<0.001
	P2		n.s.	n.s.	0.003	<0.001	<0.001	<0.001
	P3			<0.001	<0.001	<0.001	<0.001	<0.001
	P4				<0.001	<0.001	<0.001	<0.001
	P5					<0.001	<0.001	<0.001
	P6						0.005	0.030
	P7							n.s.

CMAPD: cumulative muscle activity per distance; n.s.: not significant; P1 to P8 are eight vertically oriented bipolar channels (from ventral to dorsal; see Fig 1 and Methods), with positions P1 and P2 representing TFL activity, positions P3-P5 the Gmed, and the dorsal positions P6-P8 the Gmax.

#### Walking speed-dependent effects

Mean amplitude levels were significantly higher for the fast in comparison with the normal and the slow walking speed at all positions (Fig 2A, relative difference between 10% and 40%). For position P1 and P5 to P8, the mean amplitude values were lowest for the slow walking speed. Interestingly, the results for the averaged mean amplitudes of the three individual muscles TFL (P1-P2), Gmed (P3-P5), and Gmax (P6-P8) were highly comparable to those obtained by using the equivalent of the SENIAM positions for these muscles (please see S3A and S4A Figs).

CMAPD levels showed the highest values at slow walking speed for all positions (p < 0.05 for P1-P8; Fig 2B, relative difference vs. normal: 23% to 38%; vs. fast: 16% to 41%). For the comparison of the results regarding the averaged CMAPD levels for the three individual muscles TFL, Gmed, and Gmax and their respective SENIAM positions please see S3B and S4B Figs).

### Time-dependent SEMG parameters

**Fig 3:**
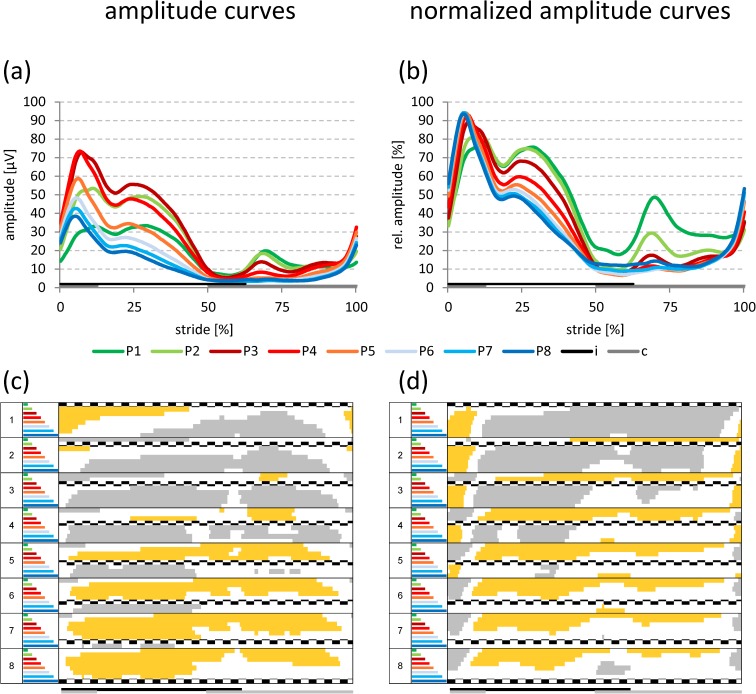
Amplitude curves at slow walking speed. Grand averaged amplitude (a) and normalized amplitude curves (b) for all electrode positions at **slow** walking speed. Data for both genders and sides are pooled. Statistically significant differences (p < 0.05; Bonferroni-Holm procedure with subsequent additional Bonferroni correction) among the different electrode positions [respective curves in (a) and (b)] are indicated in panels (c) and (d) in either gray (significantly higher values) or yellow (significantly lower values). If no colors appear in panels (c) and (d) no significant differences occurred among the positions. i: ipsilateral stance phase, c: contralateral stance phase.

**Fig 4:**
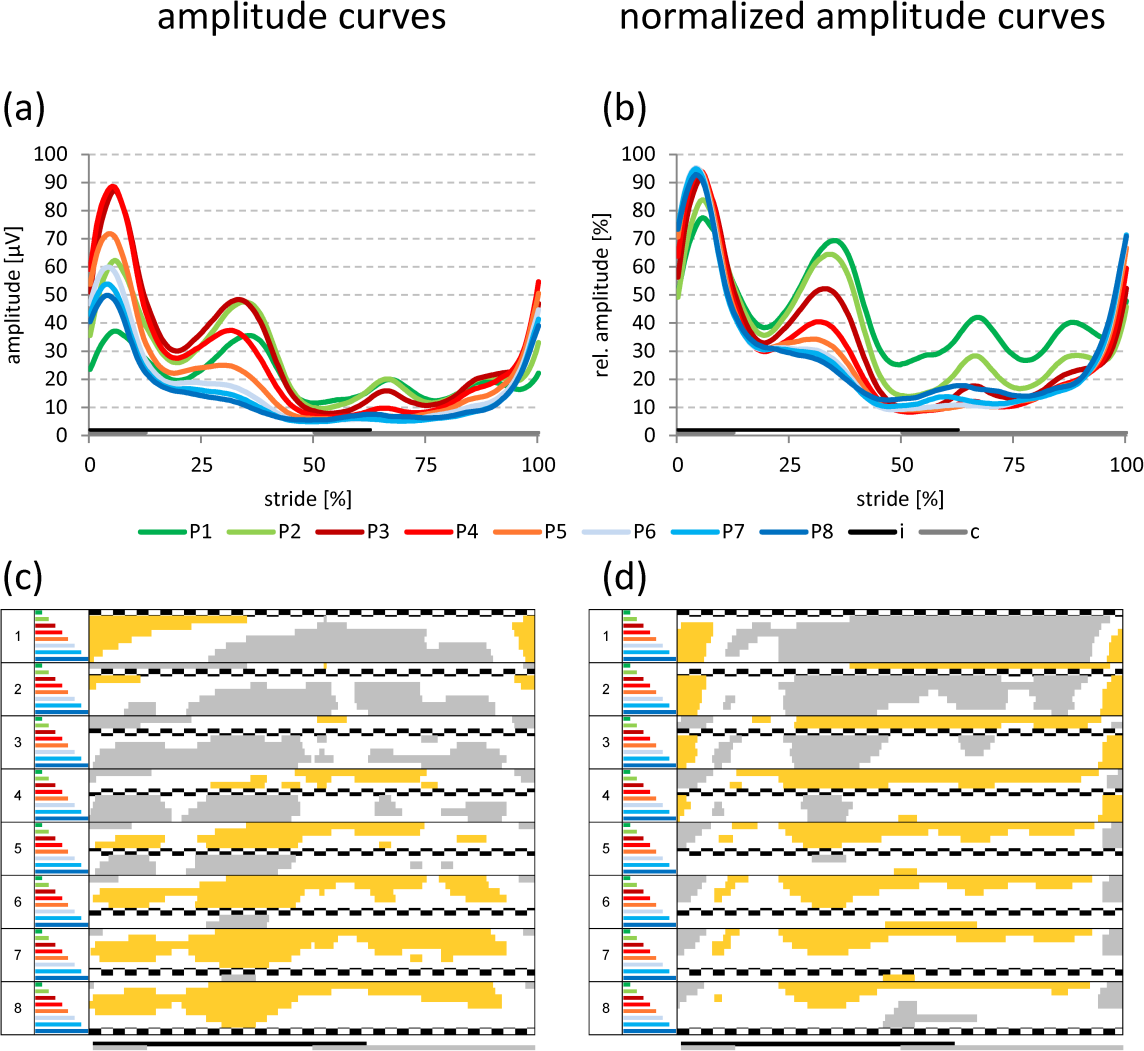
Amplitude curves at normal walking speed. Grand averaged amplitude (a) and normalized amplitude curves (b) for all electrode positions at **normal** walking speed. Data for both genders and sides are pooled. Statistically significant differences (p < 0.05; Bonferroni-Holm procedure with subsequent additional Bonferroni correction) among the different electrode positions [respective curves in (a) and (b)] are indicated in panels (c) and (d) in either gray (significantly higher values) or yellow (significantly lower values). If no colors appear in panels (c) and (d) no significant differences occurred among the positions. i: ipsilateral stance phase, c: contralateral stance phase.

**Fig 5:**
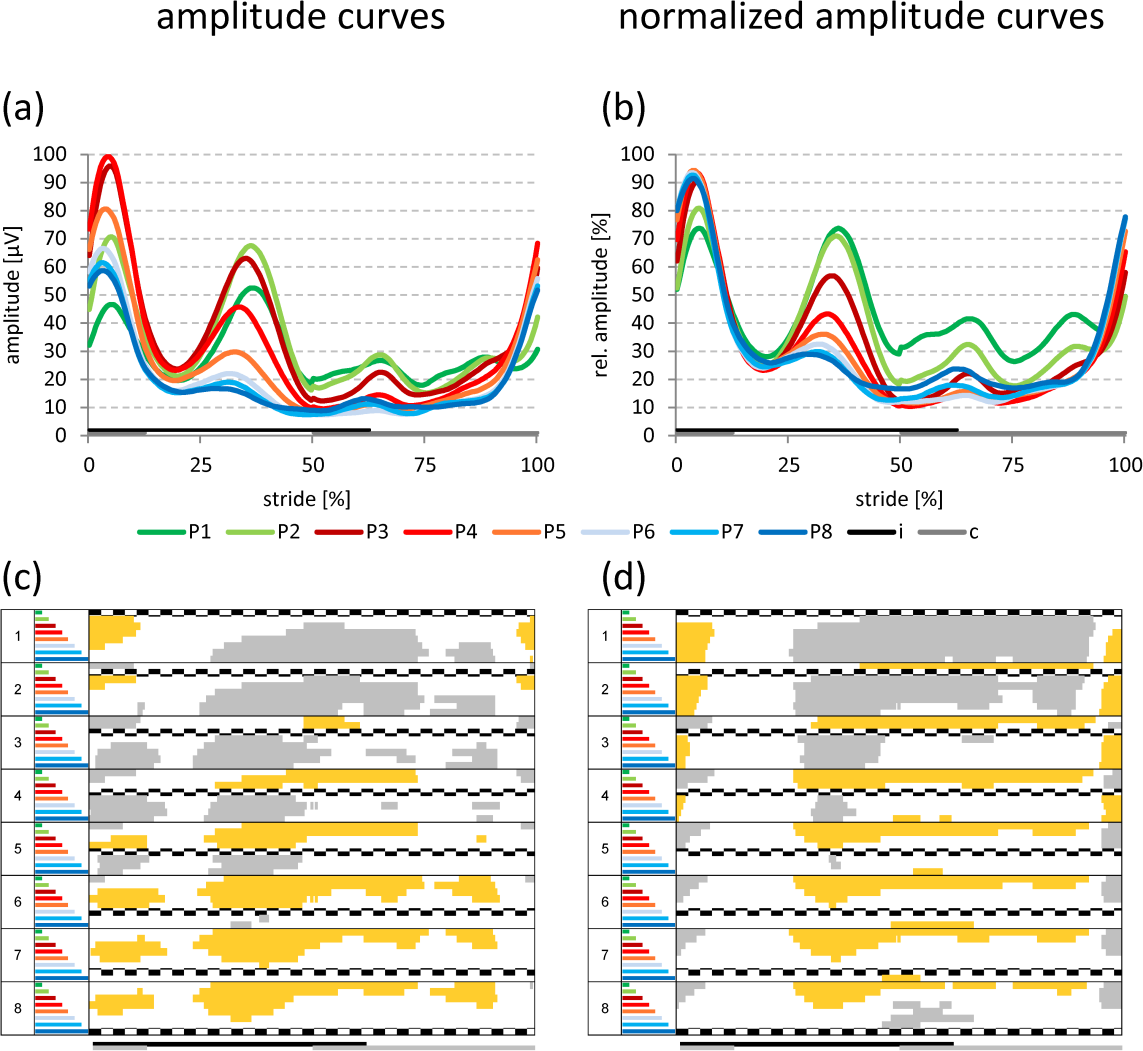
Amplitude curves at fast walkling speed. Grand averaged amplitude (a) and normalized amplitude curves (b) for all electrode positions at **fast** walking speed. Data for both genders and sides are pooled. Statistically significant differences (p < 0.05; Bonferroni-Holm procedure with subsequent additional Bonferroni correction) among the different electrode positions [respective curves in (a) and (b)] are indicated in panels (c) and (d) in either gray (significantly higher values) or yellow (significantly lower values). If no colors appear in panels (c) and (d) no significant differences occurred among the positions. i: ipsilateral stance phase, c: contralateral stance phase.

**Fig 6:**
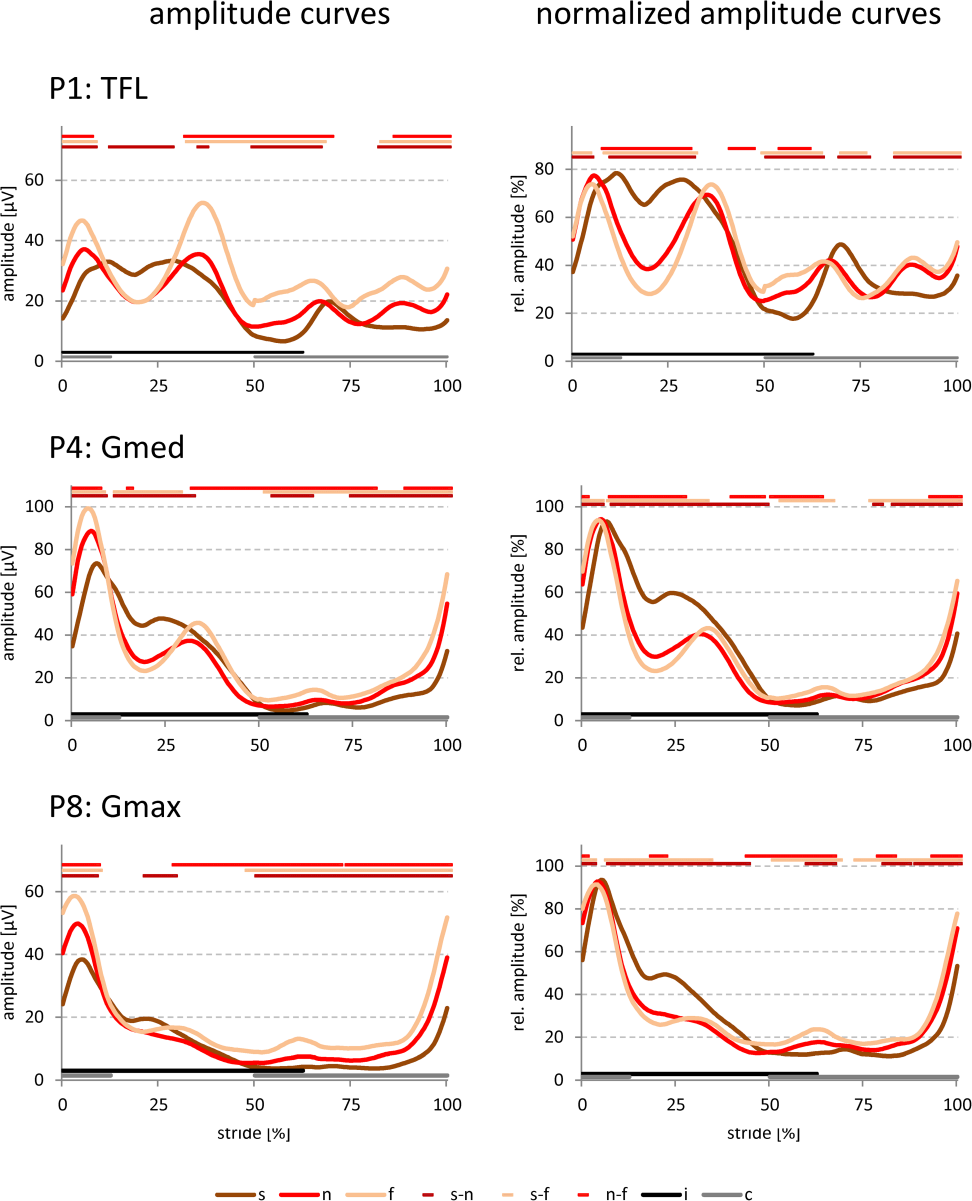
Amplitude curves for all walking speeds at positions P1, P4, and P8. Grand averaged amplitude (left column) and normalized amplitude curves (right column) for the selected electrode positions P1, P4, and P8 during **slow** (s; brown), **normal** (n; red), and **fast** (f; beige) walking speeds. Data for both genders and sides are pooled. Significant differences (p < 0.05; Bonferroni-Holm procedure with subsequent additional Bonferroni correction) among the different walking speeds are indicated by color-coded lines above the individual panels (see legend). i: ipsilateral stance phase, c: contralateral stance phase.

The curves for amplitude and normalized amplitude showed common characteristics (Figs 3–6). Irrespective of electrode position and walking speed, two amplitude peaks were identified during the stance phase, which are the SEMG equivalents of the forces required to: i) compensate for the heel strike impact; and ii) stabilize the hip (see Methods). However, the specific amplitude patterns for individual speeds and positions largely differed, in particular concerning the magnitude and the split character of the first peak, the subsequent amplitude drop between the first and the second peak, as well as the amplitude ratio between the two peaks.

#### Slow walking speed

**Fig 7:**
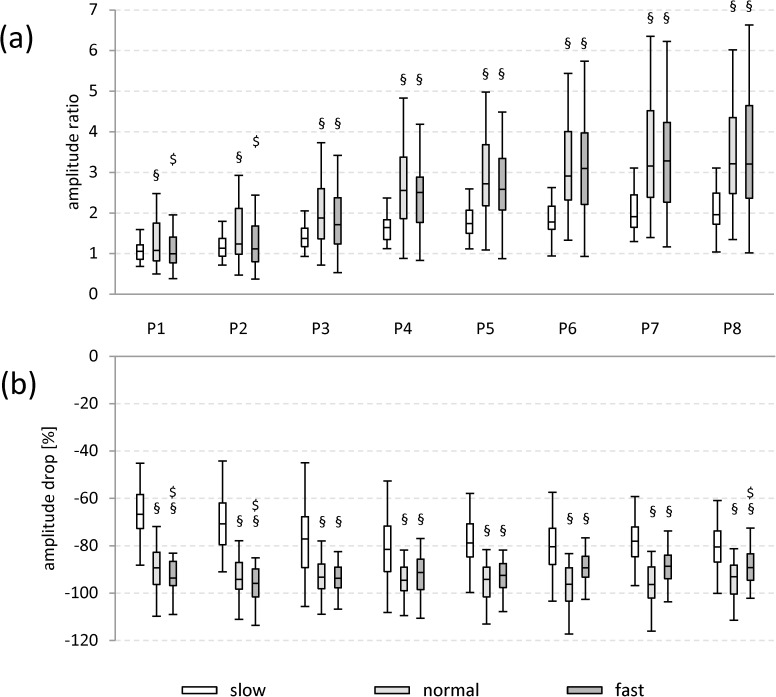
Boxplots of amplitude ratio and amplitude drop. Amplitude ratio between the two peaks (a) and amplitude drop between the two amplitude peaks (b) during the stance phase for all subjects regarding the different electrode positions (P1-P8) and walking speeds. Data are pooled for gender and side and are displayed as Boxplots. Significant differences among different walking speeds are indicated for the individual electrode positions P1-P8: § vs. slow, $ vs. normal. All p values are < 0.001 (Bonferroni corrected).

As could be expected from the mean amplitude levels (see Fig 2A), positions P2-P5 showed the highest amplitudes in peak one and two (Fig 3), resulting in statistically significant increases for positions P2-P4 in comparison to all other positions (marked in gray in Fig 3C). Also, from P1-P3/P4 the first peak was split into two separate peaks (Fig 3A and 3B). Please note that the amplitude ratio between the first and the second peak will be described below (Fig 7A).

The intermediate amplitude drop also depended on the position, i.e., it increased in magnitude from P1 (approx. -50%) to P8 (approx. -67%), if the values were calculated on an individual subject basis (see Fig 7B). Statistically significant differences were observed for positions P1-P3 in comparison to all other positions; there were no significant differences for all subsequent comparisons (Table 5).

**Table 5:** Post hoc differences for the amplitude drop among different electrode positions (including Bonferroni correction).

Walking speed		P2	P3	P4	P5	P6	P7	P8
Slow	P1	<0.001	<0.001	<0.001	<0.001	<0.001	<0.001	<0.001
	P2		<0.001	<0.001	<0.001	<0.001	<0.001	<0.001
	P3			<0.001	<0.001	0.001	0.001	<0.001
	P4				n.s.	n.s.	n.s.	n.s.
	P5					n.s.	n.s.	n.s.
	P6						n.s.	n.s.
	P7							n.s.
normal	P1	<0.001	<0.001	<0.001	<0.001	<0.001	0.001	0.002
	P2		<0.001	<0.001	0.032	n.s.	n.s.	n.s.
	P3			n.s.	n.s.	n.s.	n.s.	n.s.
	P4				n.s.	n.s.	n.s.	n.s.
	P5					n.s.	n.s.	n.s.
	P6						n.s.	n.s.
	P7							n.s.
Fast	P1	<0.001	<0.001	n.s.	n.s.	n.s.	n.s.	n.s.
	P2		n.s.	<0.001	n.s.	0.010	0.012	0.003
	P3			<0.001	n.s.	<0.001	0.001	<0.001
	P4				0.013	n.s.	n.s.	n.s.
	P5					0.002	0.043	n.s.
	P6						n.s.	n.s.
	P7							n.s.

n.s.: not significant; P1 to P8 are eight vertically oriented bipolar channels (from ventral to dorsal; see Fig 1 and Methods), with positions P1 and P2 representing TFL activity, positions P3-P5 the Gmed, and the dorsal positions P6-P8 the Gmax.

During the initial swing phase, an additional, significant amplitude peak was observed at P1-P4 (Fig 3A and 3C), which was retained for P1 and P2 in the normalized amplitude curves (Fig 3B and 3D).

#### Normal walking speed

As in the case of the slow walking speed, positions P2-P5 (peak one) and positions P1-P5 (peak two) showed the highest amplitudes in the first two peaks (Fig 4A), resulting in statistically significant increases for positions P2-P4 in comparison to all other positions (marked in gray in Fig 4C). Please note that the amplitude ratio between the first and the second peak will be described below (Fig 7A).

However, the first amplitude peak was not split anymore for any electrode position (Fig 4A). Also, the intermediate amplitude drop was considerably more pronounced for all positions, increased in magnitude from P1 (approx. -76%) to P4 (approx. -83%; Fig 7B), and then remained constant for the more dorsal positions (Figs 4B and 7B). This resulted in a clearly reduced number of statistically significant differences, which were now limited to P1 (compared to all other positions) and P2 (compared to P3-P5; Table 5).

During the swing phase, a significant double amplitude peak was observed at P1-P3 (Fig 4A and 4C), which was again only retained for P1 and P2 in the normalized amplitude curves (Fig 4B and 4D).

#### Fast walking speed

The amplitude and normalized amplitude curves at normal and fast walking speeds were very similar. However, the pronounced amplitude drop between the two peaks now reached its maximum at P3 (-84%), with a subsequent decrease in magnitude thereafter (see Fig 7B). Due to a generally high magnitude of the amplitude drop at all electrode positions (between -78% and -84%; Fig 7B), there were only randomly distributed significant differences among positions (Table 5).

#### Direct comparisons of the amplitude curves among different walking speeds

When directly addressing walking speed-dependent effects (exemplarily shown for P1 as a SENIAM equivalent for TFL; P4 for Gmed, and P8 for Gmax in Fig 6), the following common or divergent features were observed: Independent of the electrode position, the amplitude of the first peak systematically increased with higher walking speeds (Fig 6; left column). In addition, a clearly split first amplitude peak was only observed in the case of the slow walking speed. The magnitude of the subsequent amplitude drop increased from slow to fast walking speed (see Fig 6; right column). Interestingly, in the case of the slow walking speed the highest magnitude of the amplitude drop was reached at P8, whereas for the normal and fast walking speeds this occurred at P4 (Fig 6; right column).

For all walking speeds, the levels of peak one and two were comparable at P1, but clearly differed at P4 and P8, with an increase of the amplitude ratio from ventral to dorsal (Fig 6; right column; also compare with Fig 7A and S5A Fig). This ratio increase was generally lower for the slow walking speed and comparable for the other speeds (Fig 6; also compare with Fig 7A). At all positions, however, peak one occurred earlier with increasing walking speed, whereas this speed dependency was inverted for the second peak.

#### Specific curve characteristics: amplitude ratio & amplitude drop

In general, the amplitude ratio between peak one and peak two increased from P1 to P8 for all walking speeds (Fig 7A). Starting at P3, the values for the amplitude ratio increased to a higher degree for the normal and fast walking speeds, both reaching maximum values of 3.2 (Fig 7A). The amplitude ratio showed clear speed (F: 38.086, p < 0.001) and position-related effects (F: 26.856, p < 0.001), that interfered with each other (F: 7.217, p < 0.001; ANOVA).

Concerning **position-dependent**, **but speed-independent effects,** statistically significant differences were observed for positions P1-P5 in comparison to all other positions (Table 6; S5A Fig).

**Table 6:** Post hoc differences for the amplitude ratio among individual electrode positions (p values including Bonferroni correction; independent of the walking speed).

Parameter		P2	P3	P4	P5	P6	P7	P8
Amplitude ratio	P1	0.001	<0.001	<0.001	<0.001	<0.001	<0.001	<0.001
	P2		<0.001	<0.001	<0.001	<0.001	<0.001	<0.001
	P3			<0.001	<0.001	<0.001	<0.001	<0.001
	P4				n.s.	0.004	0.004	0.004
	P5					0.001	0.007	0.010
	P6						n.s.	n.s.
	P7							n.s.

n.s.: not significant; P1 to P8 are eight vertically oriented bipolar channels (from ventral to dorsal; see Fig 1 and Methods), with positions P1 and P2 representing TFL activity, positions P3-P5 the Gmed, and the dorsal positions P6-P8 the Gmax.

Interestingly, **walking speed-dependent post hoc tests** for the amplitude ratio revealed significant differences between the normal and the slow walking speed, as well as between the fast and the normal speed for the positions P1-P2, but between both the normal and fast walking speeds in comparison to the slow walking speed for positions P3-P8 (Fig 7A). Interestingly, the results for the averaged amplitude ratios of the three individual muscles TFL (P1-P2), Gmed (P3-P5), and Gmax (P6-P8) were again highly comparable to those obtained by using the equivalent of the SENIAM positions for these muscles (please see S6A and S7A Figs).

The **amplitude drop** between peak one and peak two also depended on both walking speed and position, again with the lowest magnitude occurring at slow walking speed (Fig 7B). It showed clear speed (F: 123.716, p < 0.001) and position-related effects (F: 25.943, p < 0.001), that interfered with each other (F: 13.179, p < 0.001; ANOVA).

**Position-dependent, but speed-independent effects** on the amplitude drop were highly variable among different walking speeds (S5B Fig), with heterogeneous statistical significances (Table 5).

In contrast to the amplitude ratio, **walking speed dependent post hoc tests** for the amplitude drop revealed significant differences among all walking speeds for positions P1, P2, and P8, as well as between the normal and fast walking speeds in comparison to the slow walking speed for positions P3-P7 (Fig 7B). For the comparison of the results regarding the averaged amplitude drop levels of the three individual muscles TFL, Gmed, and Gmax and their respective SENIAM positions please see S6B and S7B Figs).

## Discussion

The current report regards a systematic study on the activation pattern of individual superficial hip muscles in healthy controls. Within the limitations discussed below, it represents the first report on the detailed spatial SEMG characterization of superficial hip muscle activation during walking and results in the novel interpretation that this activation pattern may reflect finely tuned coordination patterns of neighboring muscles and muscle segments (in the sense of an 'intermuscular functional unit') rather than the independent activation of individual muscles. This interpretation particularly takes into consideration the existence of: i) anatomically defined individual muscles with specific functions (i.e., TFL, Gmed, and Gmax; [3]); ii) separately innervated muscle subregions [8, 9]; and iii) functional redundancy and/or collaboration of different muscles (or their subregions [11]).

Concerning the speed dependency of this 'intermuscular unit', the SEMG data at slow speed showed a substantially elevated energy expenditure per distance (CMAPD) and an augmented tonic behavior during the stance phase (i.e., a reduced amplitude drop), strongly indicating that this speed is unfavorable for muscle efficacy and metabolic supply [35]. In addition, there was a clear position dependency of the data (inter-dependent with the walking speed), indicating a highly differential, relative importance of different regions of the gluteal 'intermuscular unit' for the function of the hip joint during locomotion.

This study also confirmed the existence of two separate peaks in the stance phase of the gait cycle, their relative position (peak one: approx. 5%; peak two: approx. 35%), and their speed dependency [3, 4, 11, 33, 36]. Our study additionally confirmed a strong influence of the electrode position on all time-independent parameters, analogous to data using either invasive needle EMG for the Gmed [11] or SEMG for various other muscles [37–39].

### Influence of the walking speed

#### Time-independent parameters

For the TFL and the Gmed (P1-P5; compare with S3 Fig), only the CMAPD, but not the mean amplitude, differentiated the self-selected, slow speed (3.2 km/h), from the other, energetically optimized speeds (i.e., normal = 5.3 km/h; fast = 6.3 km/h; [36, 40]). This is compatible with the notion that, for the non-fatigued muscle, the mean amplitude represents muscular effort independent of time and traveled distance, whereas the CMAPD reflects the energy expenditure for a given traveled distance (e.g., 1 km; [35]).

#### Time-dependent parameters

For the slow walking speed, the first peak during the stance phase was split into two separate peaks for the TFL (i.e., P1 and P2) and Gmed positions (i.e., P3-P5). This split first peak, likely representing the heel and the forefoot contact during the load response, has not been described in detail in other studies [3, 4, 11] and was probably deciphered in the present study due to the high time resolution of 0.5% of the normalized stride. The split character of the first peak could not be resolved anymore for the normal and fast walking speed, possibly due to the shortened period for the load response [3].

The present study also showed details of the **amplitude drop** between the two peaks, representing a period of reduced muscle activation and corresponding to the decline phase just after the weight acceptance peak in the vertical ground reaction force [33, 41]. This level of detail was also likely due to the high time and amplitude resolution.

This amplitude drop became more pronounced with increasing speed (please see Figs 3–6), also in good correspondence with the speed-dependent changes of the intermediate force valley in the vertical ground reaction force (named F2; 33).

When regarding the **amplitude ratio** between peak one and two for Gmed and Gmax in a position-independent fashion, the normal and fast walking speeds reached 50% higher values than the slow walking speed (see S5A Fig). This is again compatible with the corresponding speed-dependent changes in the two peaks of the vertical ground reaction force [42, 43], reflecting the body weight dropping onto and moving across the supporting foot [33]. Interestingly, the TFL (i.e., P1 and P2) did not show such a behavior, indicating that for this muscle the underlying functional importance of the two peaks may be mostly independent of the walking speed.

The second of the two peaks during the **swing phase** (present in the TFL electrodes P1 and P2 and reflecting stabilization of the leg during the preparation of the next heel strike [4, 29]) also became considerably more pronounced with increasing speed. This is in agreement with an increased necessity for the fine-tuning of the decelerating activity of the hamstring muscles by the TFL [3].

### Influence of the electrode position

#### Time-dependent parameters

For all walking speeds, the amplitude of peak one steeply increased from P1 to P3/P4 and then gradually decreased to P8 (in analogy to the mean amplitude; see Fig 2A). As a consequence, P2-P5 showed the highest amplitudes in peak one, resulting in statistically significant increases for P2-P4 in comparison to virtually all other positions (see Figs 3–6).

Due to the temporal coincidence of peak one with the loading response, this finding indicates a pivotal role of these ventral hip muscle regions (P2-P4; i.e., 1.25 to 6.25 cm ventral to the line between iliac crest and greater trochanter) for the compensation of the heel and forefoot contact during the initial stance phase [33].

This picture of steeply increasing amplitudes from P1 to P3/P4 considerably changed in peak two, resulting in an altered order of the electrode positions (compare with Figs 3A to 5A). Also, the normalized amplitude values in peak two (and thus the amplitude ratio between peak one and two) continuously decreased towards the more dorsal positions (compare with Figs 3B to 5B). This finding strongly indicates that the more ventral parts of the gluteal 'intermuscular functional unit' (P1-P5; i.e. TFL and Gmed) are specifically important for the stabilization of the hip against its contralateral subsidence during this part of the ipsilateral stance phase [1].

The amplitude drop during the stance phase showed an interesting position dependency (increase from ventral to dorsal), in particular visible for the slow speed. This may result in a nutrition disadvantage for the ventral region during the stance phase at this speed, since a decreased amplitude drop reflects a more tonic, less alternating activation pattern, potentially more susceptible to reduced muscular blood supply. However, this may be a special situation occurring only at slow walking speed, which differs from the other walking speeds by a proportionally longer stance phase [6, 33].

### Neuro-functional considerations

Muscles and their subunits never act as independent structures, but always in a functionally driven manner to reach "global goals" [22, 23, 44]. This was proven by the identification of coupled activation patterns of spinal cord neurons that consist of five major components [24] and is further supported by the fact that for the investigated muscles the supplying nerves also show a large overlap with respect to the nerve roots [45, 46].

In the present study, all analyzed parameters showed very similar characteristics with subtle differences between electrode positions. Interestingly, the differences changed across the parameters: i) mean amplitude and CMAPD continuously increased from P1 to P3 (P4), but decreased again towards the more dorsal positions; ii) the amplitude ratio instead continuously increased from ventral to dorsal; iii) the amplitude drop also increased from ventral to dorsal, but reached a plateau at positions P4 (P5) to P8. This also applied to the two peaks during initial and mid stance, which showed a continuous time shift from dorsal to ventral.

In summary, the muscles of the gluteal region behaved like a functionally coupled, finely tuned but spatially enlarged ensemble or 'intermuscular unit' rather than single, independently acting muscles. This is also in agreement with the modern, functionally based understanding of coordinated trunk muscle activation that has resulted in a widely accepted, functionally based classification [47].

### Limitations

Obviously, there exists a conflict between the fixed electrode configuration and the variable anatomical conditions among subjects. We tried to minimize this by centrally aligning the electrode strips, which cut the spatial variability into half. Therefore, only the most ventral position P1, the mid-positions P4 and P5, and the most dorsal position P8 can reliably assigned to TFL, Gmed, and Gmax, respectively. The remaining positions P2 & P3 and P6 & P7 may contain cross-talk information from the respective neighboring muscles, due to inter-individual variation of pelvic bone and soft tissue anatomy in contrast to the defined geometry of the electrode positions. Cross-talk can be considerably reduced by the application of spatial filters [48], but quantification of its importance requires exact positioning of the electrodes on the target muscles and their selective activation, which is virtually impossible for these highly agonistic muscles. Although the monopolar montage applied in the present study would theoretically allow such spatial filtering, the conflict between individual anatomy and electrode geometry would thus remain. However, there is an excellent agreement between the present data and those of Semciw et al., who performed intramuscular EMG recordings, which are not prone to crosstalk and whose exact positioning was further verified by ultrasound [30]. We thus believe that, although cross-talk principally remains a relevant limitation, it does not fundamentally question the current results showing spatially differentiated activation patterns of the gluteal region [11].

On the other hand, if considering the investigated region as an ensemble of a partial functionally redundant 'intermuscular unit' with gradual spatial organization, but functionally adapted behavior, the inherent crosstalk information may help to obtain a smoothed picture of its organization with respect to amplitude levels and timing [22].

In the present study, position-dependent amplitude dampening based on regional differences of the subcutaneous fat layer thickness cannot be totally excluded. In fact, the thickest fat layer has been described at the central to dorsal gluteal regions [49], reflected in positions P5-P8 of our study. However, own published data demonstrated almost identical SEMG amplitudes without regional differences for the recommended electrode positions of TFL, Gmed, and Gmax (www.seniam.org) across various MVC tasks [26], also in good agreement with respective data by Kaneda and Colleagues [50]. In addition, local skinfold measurements (reflecting differences in subcutaneous fat layer thickness) showed a poor correlation with the inter-individual variance of the SEMG amplitude [51]. Thus, local differences of the fat layer among positions P1 to P8 are not likely to have systematically biased the observed amplitude profiles in the present study.

Another factor potentially influencing the amplitude levels is the deviation of the fiber direction of the respective muscle portions from the vertically oriented, bipolar electrode channels [29, 30]. Since the amplitude levels in the present study were always highest at P3 (with at least partially oblique muscle fibers), and not at positions P4 and/or P5 (likely with predominantly parallel muscle fibers), variations of the fiber direction also do not likely explain the amplitude profiles in the present study.

### Conclusions

The mode of action of the superficial gluteal muscles appears to reflect an 'intermuscular functional unit'. This is supported by the fact that the analyzed SEMG parameters identified only continuous changes for the separate electrodes across this region. The ventral parts of this intermuscular unit (represented by the electrode position P2 to P4) are of utmost importance during human walking. This is demonstrated by all time-independent and time-dependent parameters and includes all walking speeds, with minor limitations during non-physiologically slow walking speed. These findings are likely relevant for both the physiological function of this intermuscular unit, i.e., its role during heel contact, hip stabilization, and swing phase, and for the surgical access during standard (ventral or dorsal) or minimally-invasive hip replacement. The measured finely tuned and highly coordinated activation is of clear clinical relevance in view of the large, continuously increasing number of hip joint replacements [13] and in view of the mean age of the present cohort, which is representative for the age group of patients undergoing total hip arthroplasty.

A more spatially resolved SEMG investigation of this region, e.g., by expanding the currently applied single bipolar electrode position for the Gmed by two additional bipolar electrode positions (one horizontally aligned to the SENIAM position at mid-distance between ASIS and greater trochanter; the other one at the same distance, but in the dorsal direction), can thus be suggested in order to differentiate regional activation patterns of this muscle during walking. This may support the development of novel, fine-tuned strategies for diagnostics, (surgical) therapy, and rehabilitation, taking into consideration both the suitability of selected walking speeds and the individual fatigue level.

## Supporting information

S1 Fig Images of a cadaver specimen. Representation of the spatial relationship between the gluteal muscles (left column) and the electrode strips (right column). The black pinheads mark the landmarks greater trochanter and the iliac crest, the red pinhead is located at mid-distance between these two anatomical landmarks. The lines of orange and blue pinheads mark the dorsal border of the TFL and the ventral border of the Gmax, respectively. This comparison shows that electrode P1 and P2 are located above the TFL, electrodes P3 to P5 above the Gmed, and electrodes P6 to P8 above the Gmax.
(TIFF)

S2 Fig Boxplots of speed-averaged mean amplitude and CMAPD. Values of mean amplitude (a) and CMAPD (b) for all subjects regarding the different electrode positions. The results of the respective post hoc tests are presented in Table 4.
(TIFF)

S3 Fig Boxplots of position-averaged mean amplitude and CMAPD. Values for TFL (P1 and P2), Gmed (P3-P5), and Gmax (P6-P8) of mean amplitude (a) and CMAPD (b) for all subjects. Significant differences among the different walking speeds are indicated for the individual muscles: § vs. slow, $ vs. normal. All p values are < 0.05 (Bonferroni corrected).
(TIFF)

S4 Fig Boxplots of the SENIAM-equivalent positions for mean amplitude and CMAPD. Values of the SENIAM equivalent positions for TFL (P1), Gmed (P4-P5), and Gmax (P8) of mean amplitude (a) and CMAPD (b) for all subjects. Significant differences among the different walking speeds are indicated for the individual muscles: § vs. slow, $ vs. normal. All p values are < 0.05 (Bonferroni corrected).
(TIFF)

S5 Fig Boxplots of speed-averaged amplitude ratio and amplitude drop. Values of the amplitude ratio between the two peaks (a) and the amplitude drop after the first peak (b) during the stance phase for all subjects regarding the different electrode positions. The results of the respective post hoc tests for the amplitude ratio are presented in Table 6.
(TIFF)

S6 Fig Boxplots of position-averaged amplitude ratio and amplitude drop. Values for TFL (P1 and P2), Gmed (P3-P5), and Gmax (P6-P8) of the amplitude ratio between the two peaks (a) and the amplitude drop after the first peak (b) during the stance phase for all subjects. Significant differences among the different walking speeds are indicated for the individual muscles: § vs. slow, $ vs. normal. All p values are < 0.05 (Bonferroni corrected).
(TIFF)

S7 Fig Boxplots of the SENIAM-equivalent positions for amplitude ratio and amplitude drop. Values for TFL (P1), Gmed (P4-P5), and Gmax (P8) of the amplitude ratio between the two peaks (a) and the amplitude drop after the first peak (b) during the stance phase for all subjects. Significant differences among the different walking speeds are indicated for the individual muscles: § vs. slow, $ vs. normal. All p values are < 0.05 (Bonferroni corrected).
(TIFF)

S1 Text Detailed description of the applied statistical calculations for the analysis of amplitude curves. (DOCX)
