# Supplementary figures and images for: Detailed spatial characterization of superficial hip muscle activation during walking: A multi-electrode surface EMG investigation of the gluteal region in healthy older adults
Source: PLoS One. 2017 Jun 5;12(6):e0178957. doi: 10.1371/journal.pone.0178957 (PMC5459501; doi:10.1371/journal.pone.0178957)

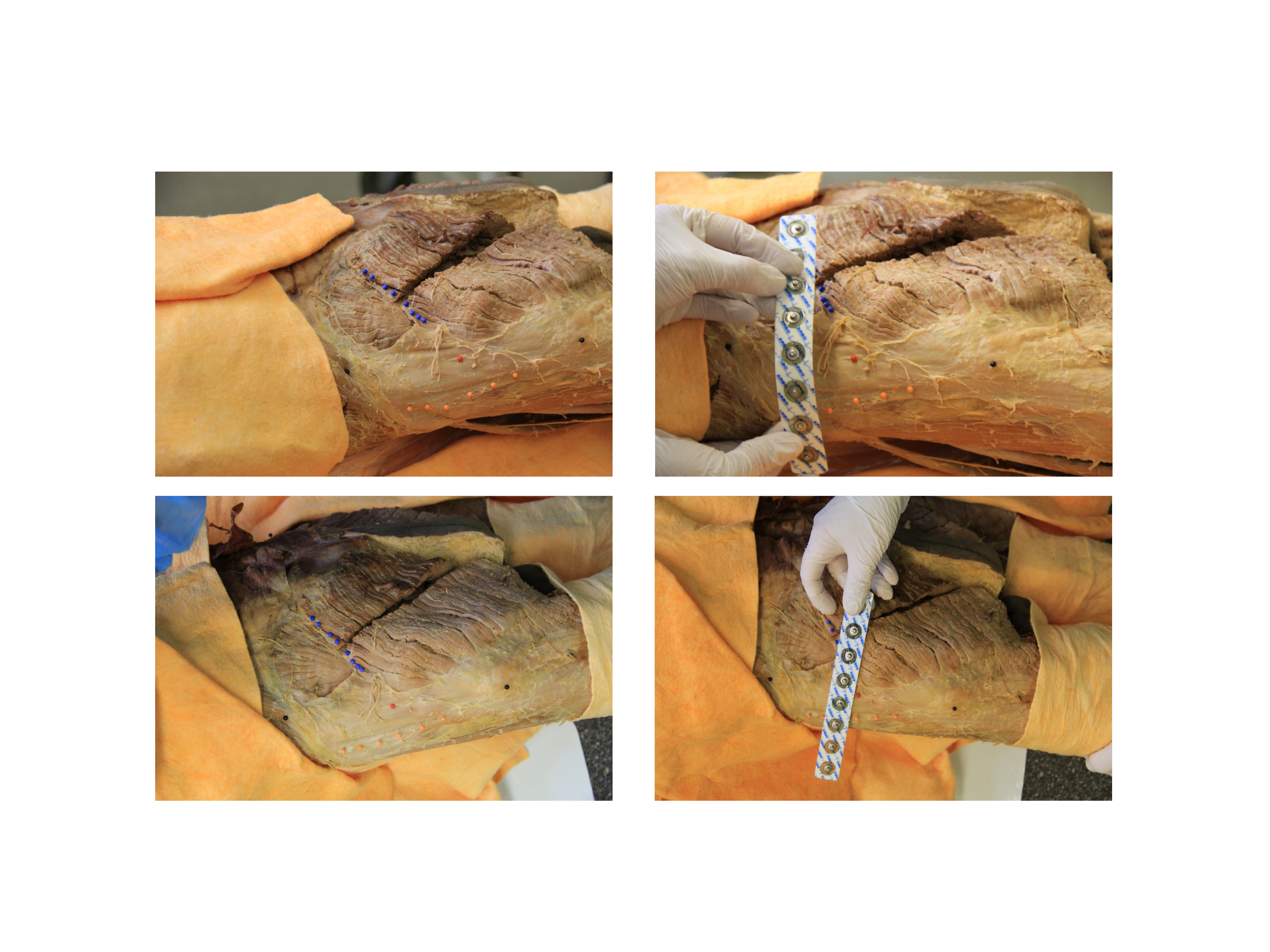

Supplement: S1 Fig — Representation of the spatial relationship between the gluteal muscles (left column) and the electrode strips (right column). The black pinheads mark the landmarks greater trochanter and the iliac crest, the red pinhead is located at mid-distance between these two anatomical landmarks. The lines of orange and blue pinheads mark the dorsal border of the TFL and the ventral border of the Gmax, respectively. This comparison shows that electrode P1 and P2 are located above the TFL, electrodes P3 to P5 above the Gmed, and electrodes P6 to P8 above the Gmax. (TIFF) [file pone.0178957.s001.tiff]

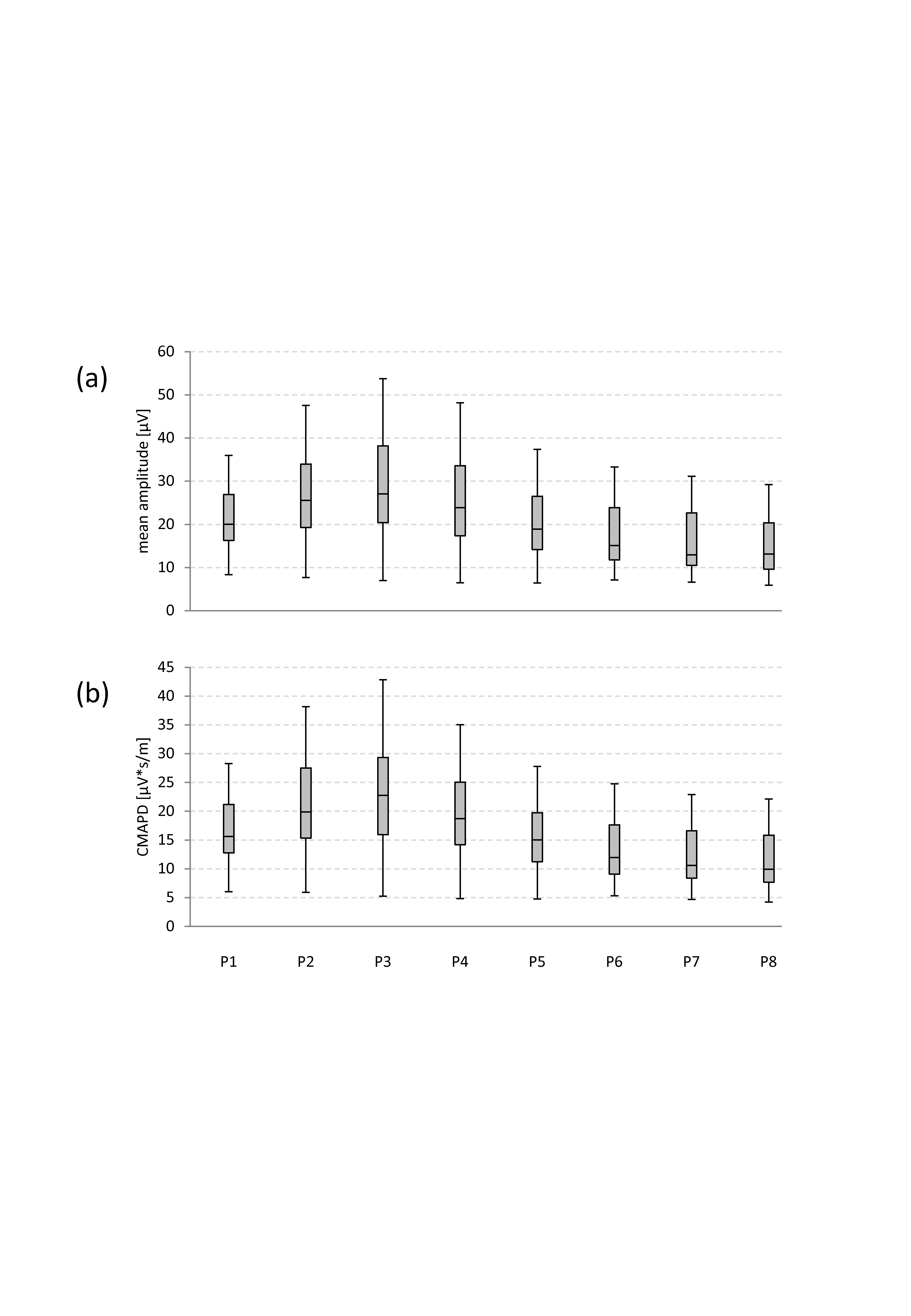

Supplement: S2 Fig — Values of mean amplitude (a) and CMAPD (b) for all subjects regarding the different electrode positions. The results of the respective post hoc tests are presented in Table 4. (TIFF) [file pone.0178957.s002.tiff]

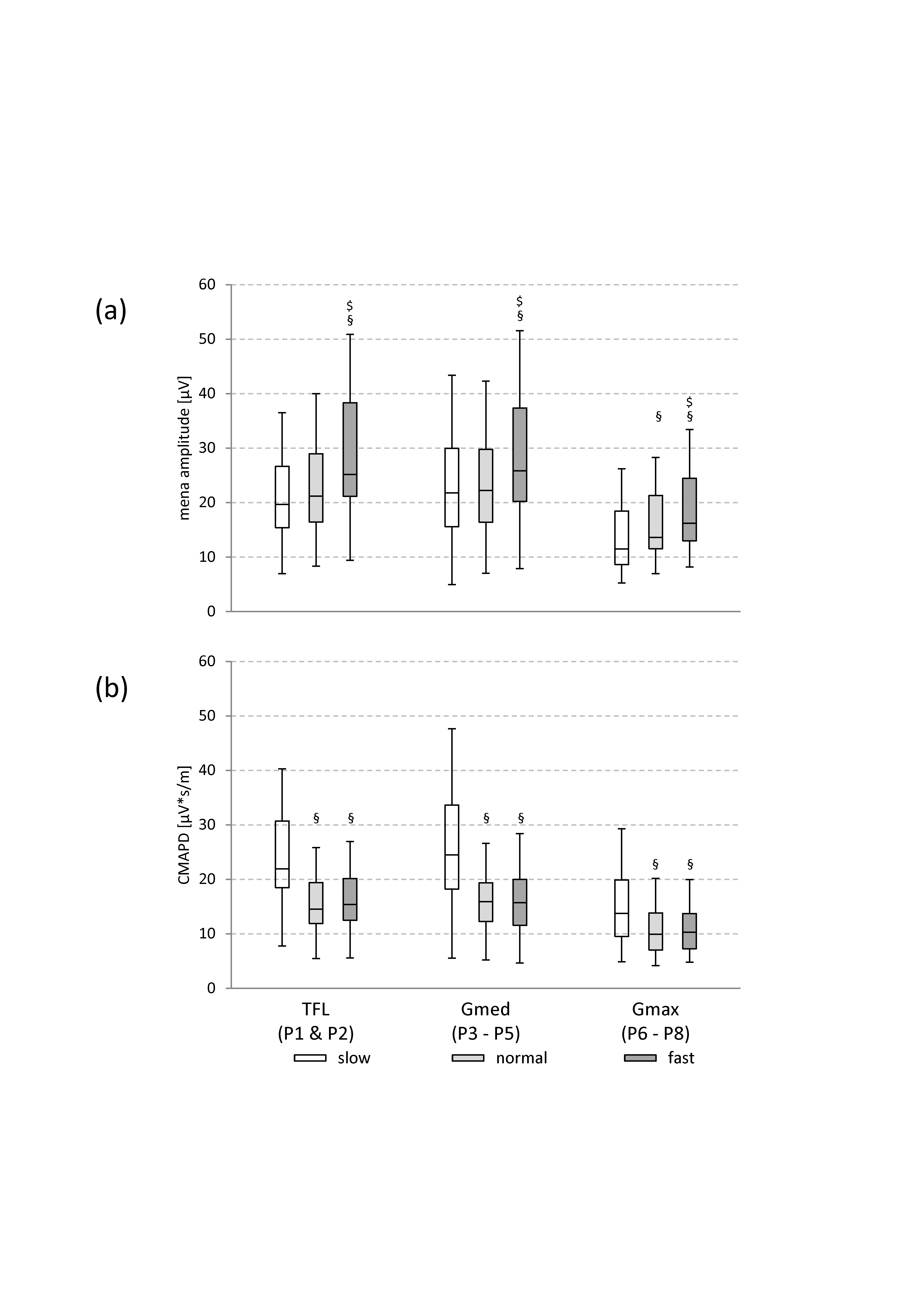

Supplement: S3 Fig — Values for TFL (P1 and P2), Gmed (P3-P5), and Gmax (P6-P8) of mean amplitude (a) and CMAPD (b) for all subjects. Significant differences among the different walking speeds are indicated for the individual muscles: § vs. slow, $ vs. normal. All p values are < 0.05 (Bonferroni corrected). (TIFF) [file pone.0178957.s003.tiff]

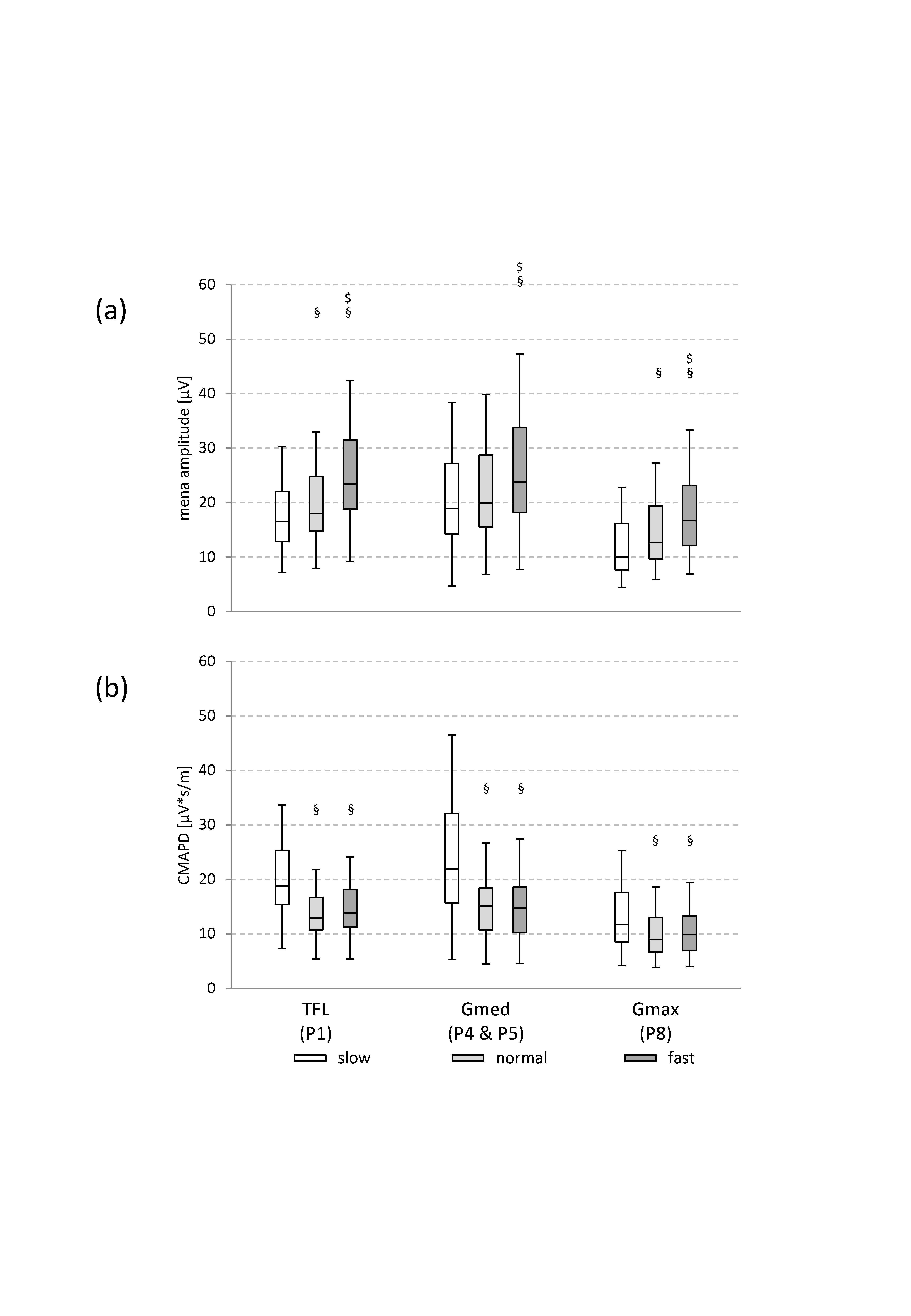

Supplement: S4 Fig — Values of the SENIAM equivalent positions for TFL (P1), Gmed (P4-P5), and Gmax (P8) of mean amplitude (a) and CMAPD (b) for all subjects. Significant differences among the different walking speeds are indicated for the individual muscles: § vs. slow, $ vs. normal. All p values are < 0.05 (Bonferroni corrected). (TIFF) [file pone.0178957.s004.tiff]

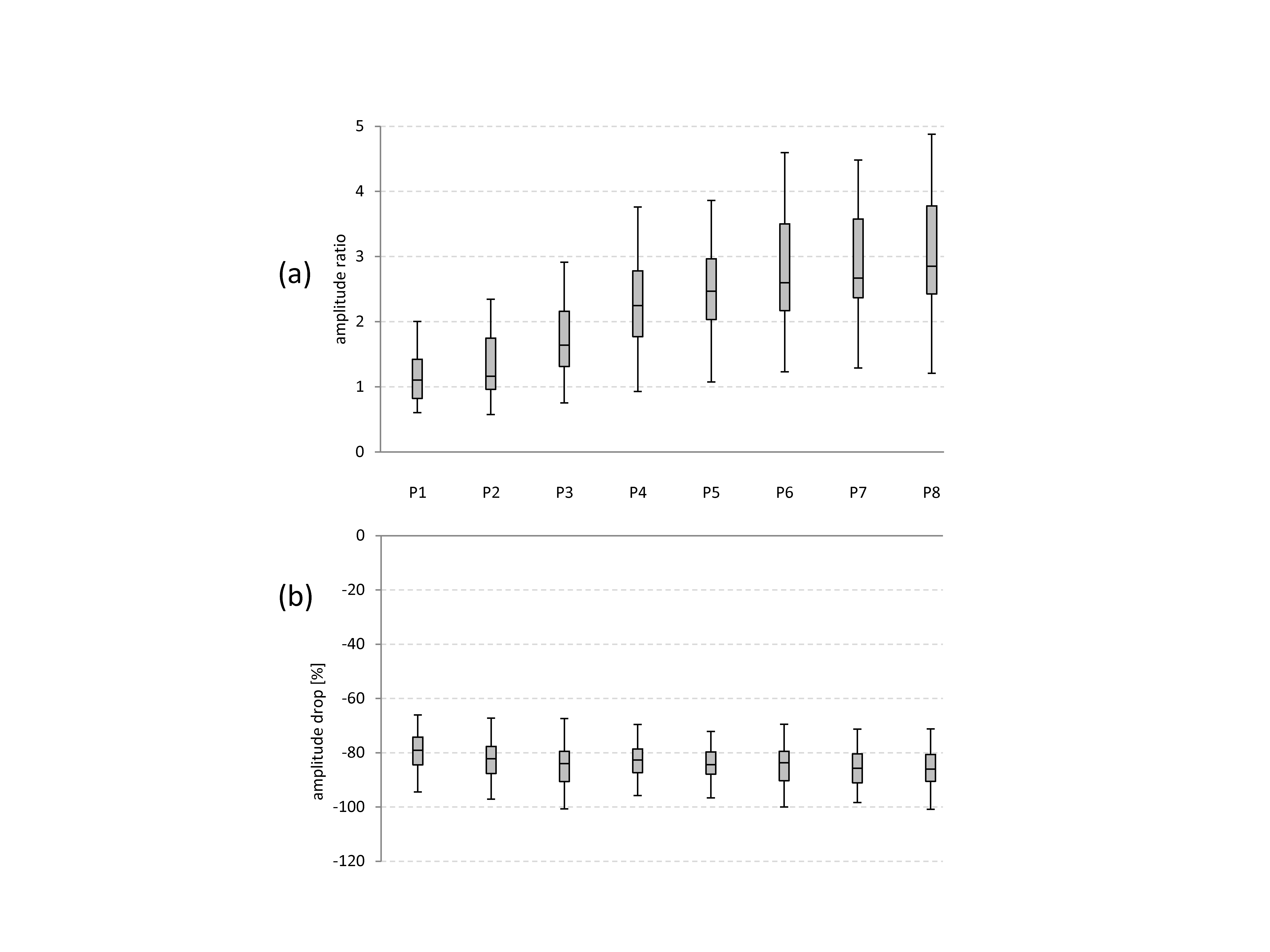

Supplement: S5 Fig — Values of the amplitude ratio between the two peaks (a) and the amplitude drop after the first peak (b) during the stance phase for all subjects regarding the different electrode positions. The results of the respective post hoc tests for the amplitude ratio are presented in Table 6. (TIFF) [file pone.0178957.s005.tiff]

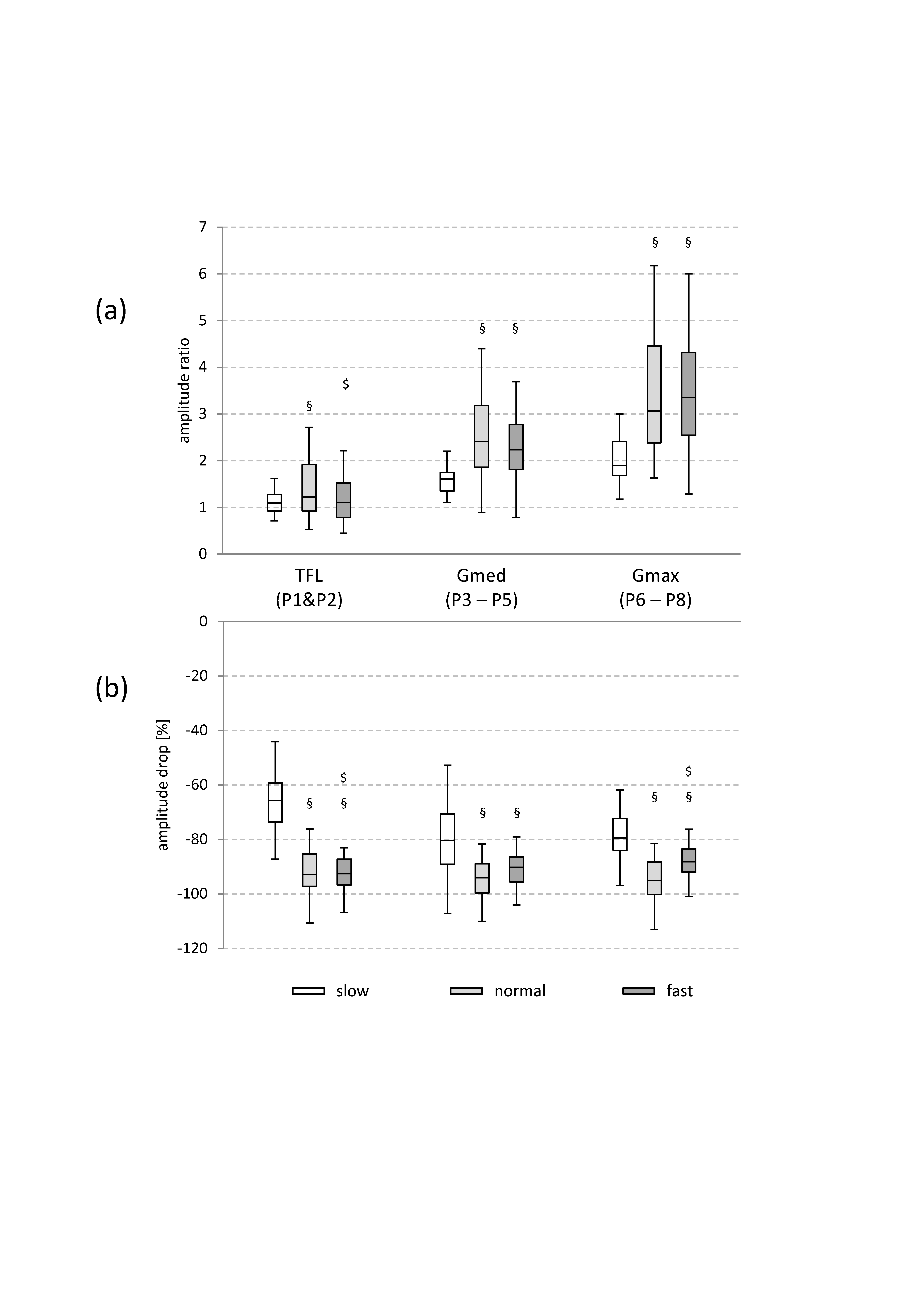

Supplement: S6 Fig — Values for TFL (P1 and P2), Gmed (P3-P5), and Gmax (P6-P8) of the amplitude ratio between the two peaks (a) and the amplitude drop after the first peak (b) during the stance phase for all subjects. Significant differences among the different walking speeds are indicated for the individual muscles: § vs. slow, $ vs. normal. All p values are < 0.05 (Bonferroni corrected). (TIFF) [file pone.0178957.s006.tiff]

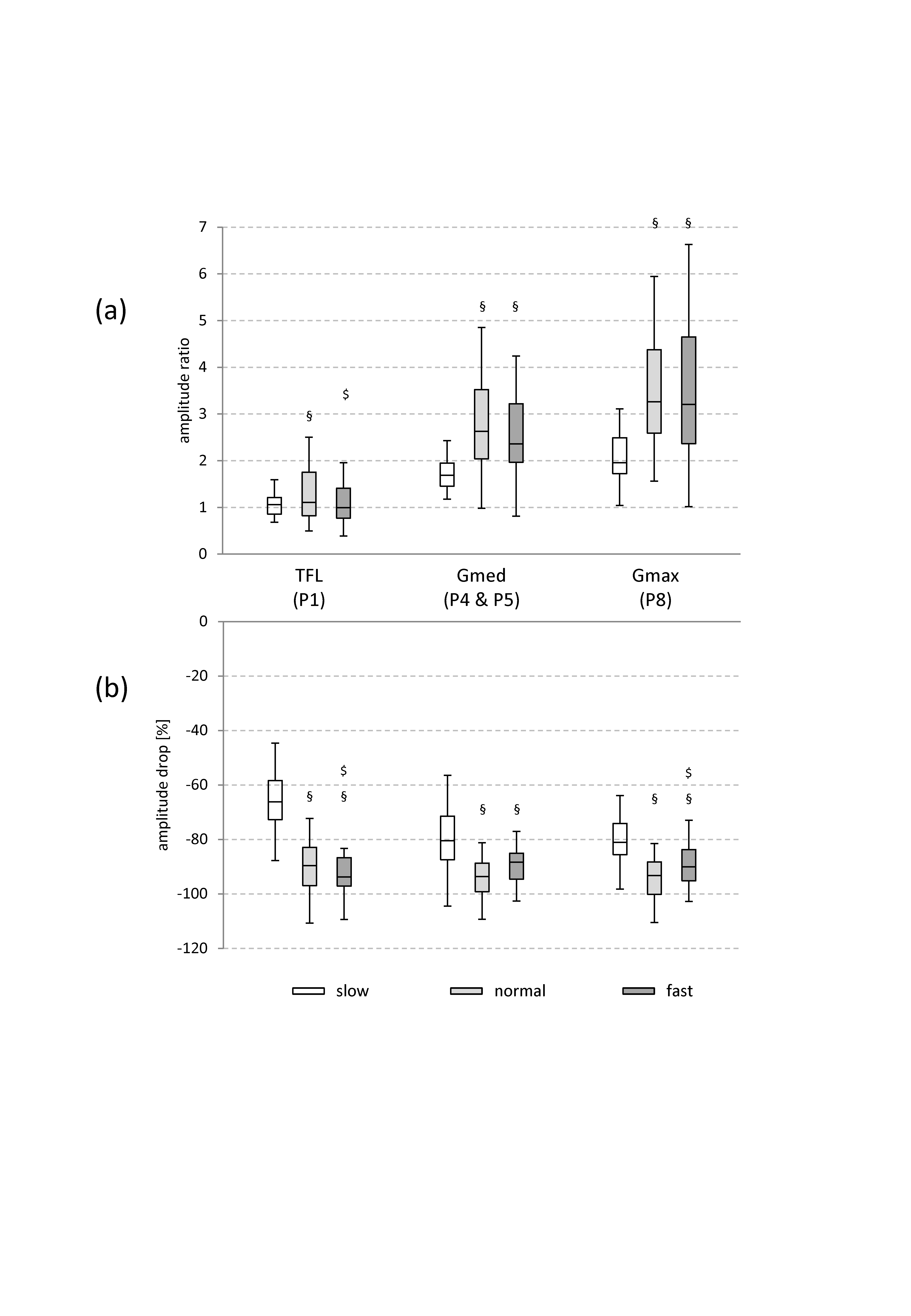

Supplement: S7 Fig — Values for TFL (P1), Gmed (P4-P5), and Gmax (P8) of the amplitude ratio between the two peaks (a) and the amplitude drop after the first peak (b) during the stance phase for all subjects. Significant differences among the different walking speeds are indicated for the individual muscles: § vs. slow, $ vs. normal. All p values are < 0.05 (Bonferroni corrected). (TIFF) [file pone.0178957.s007.tiff]
