# Supplementary material for: Detailed spatial characterization of superficial hip muscle activation during walking: A multi-electrode surface EMG investigation of the gluteal region in healthy older adults
Source: PLoS One. 2017 Jun 5;12(6):e0178957. doi: 10.1371/journal.pone.0178957 (PMC5459501; doi:10.1371/journal.pone.0178957)
Supplement: S1 Text — (DOCX) [file pone.0178957.s008.docx]

### Detailed description of the applied statistical calculations for the analysis of amplitude curves

The k = 201 single time points of the time-normalized SEMG data had to be individually compared among the respective groups. Let x = (x_k1_, …, x_k201_) and y = (y_k1_, …, y_k201_) denote the two vectors (groups) to be compared and (µ_xk1_, …, µ_xk201_) and (µ_yk1_, …, µ_yk201_) be the respective means. The calculated p values for the rejection of the null hypotheses H_k1_: µ_xk1_ = µ_yk1_, …, H_k201_: µ_xk201_ = µ_yk201_ were ranked in ascending order where H_1_ denotes the smallest and H_201_ the largest p level. The p value for the rejection of each individual null hypothesis was then compared to its required α level (e.g., α_1_/k (0.05/201 = 0.000249) for H_1_; α_2_/(k-1) (0.05/200 = 0.00025) for H_2_ etc.). As long as a given p was smaller than its required α level, the respective null hypothesis was rejected and the algorithm continued until p exceeded the required α level. At this point, the differences among the different groups were no longer considered statistically significant.
